# Emotion and Value in the Evaluation of Medical Decision-Making Capacity: A Narrative Review of Arguments

**DOI:** 10.3389/fpsyg.2016.00765

**Published:** 2016-05-26

**Authors:** Helena Hermann, Manuel Trachsel, Bernice S. Elger, Nikola Biller-Andorno

**Affiliations:** ^1^Institute of Biomedical Ethics and History of Medicine, University of ZurichZurich, Switzerland; ^2^Institute for Biomedical Ethics, University of BaselBasel, Switzerland; ^3^Center for Legal Medicine, University of GenevaGeneva, Switzerland

**Keywords:** competence, decision-making capacity, emotion, value, self-determination, informed consent, review

## Abstract

Ever since the traditional criteria for medical decision-making capacity (*understanding, appreciation, reasoning, evidencing a choice*) were formulated, they have been criticized for not taking sufficient account of emotions or values that seem, according to the critics and in line with clinical experiences, essential to decision-making capacity. The aim of this paper is to provide a nuanced and structured overview of the arguments provided in the literature emphasizing the importance of these factors and arguing for their inclusion in competence evaluations. Moreover, a broader reflection on the findings of the literature is provided. Specific difficulties of formulating and measuring emotional and valuational factors are discussed inviting reflection on the possibility of handling relevant factors in a more flexible, case-specific, and context-specific way rather than adhering to a rigid set of operationalized criteria.

## Introduction

Patients’ decision-making capacity or competence (DMC) is the gatekeeping element establishing the role of patient choices in medical decisions. In view of the legal and ethical implications, the concept has been intensely investigated both theoretically and empirically over several decades.

One long-standing debate concerns the definition of relevant criteria of DMC, in terms of mental abilities. In the 1990s, great advances were made by Grisso and Appelbaum (1998), who distilled and systemized relevant mental abilities on the basis of United States case law (Appelbaum and Grisso, 1995). This extensive work yielded the four traditional criteria that have remained influential ever since: (1) *understanding* refers to the ability to comprehend treatment-related information, such as information about the present disorder, treatment options, and related risks and benefits; (2) *appreciation* refers to the ability to acknowledge that one is suffering from a particular disorder (i.e., insight into the disorder). It also refers to the ability to recognize the consequences for oneself of the disorder and of potential treatment options, including the ability to acknowledge that treatment could be beneficial (i.e., insight into the necessity of treatment); (3) *reasoning* refers to the ability to manipulate information rationally, using logic to compare the risks and benefits of treatment alternatives; and (4) *evidencing a choice* refers to the ability to communicate a choice (Grisso and Appelbaum, 1998).

While these standards have some validity and have significantly helped to improve DMC evaluations, they have been and continue to be challenged. Critics characterize the traditional approach as too cognitive and/or too procedural in failing to take proper account of non-cognitive factors and/or substantive elements (e.g., Charland, 1998b; Banner, 2013). Specifically, critics advocate for fuller acknowledgment of emotional factors and values within DMC evaluations.

Various, sometimes overlapping or even conflicting arguments have been advanced in favor of this claim. However, the literature fails to provide a comprehensive understanding of the significance of emotions and values in DMC evaluation, making any significant advance in this matter difficult. To overcome this barrier, the present paper provides, first, a nuanced and structured overview of the medical ethics literature with regard to existing arguments for stronger recognition of emotions and values in DMC evaluations. Second, a broader reflection and discussion on the findings of the literature are provided. The aim of this overview is to advance the debate, to stimulate further development, and last, but not least to inform clinicians seeking for stronger theoretical embedding of their clinical observations.

## Method And Framework

The paper provides a narrative review of arguments. Publications were considered that place a primary focus on conceptual analysis concerning the role of emotion and value in DMC. Arguments were extracted while looking for argument variation and saturation.

Subsequently, arguments were assorted into four categories along two dimensions: (a) domain (*emotion* vs. *value*), and (b) account (*procedural* vs. *substantive*). The categorization aims to foster conceptual clarity and to help identify potential research gaps. **Table 1** summarizes the findings.

**Table 1:** Arguments for acknowledging emotion and value in DMC.

Emotion	Value
Substantive account (content-laden)	• Emotions provide crucial information and are essential in decision making –Full appreciation requires emotion –Strong emotional avoidance/denial is problematic • Affective arousal derails cognitive functions and overpowers rational thought Too little or too much of emotional engagement is problematic • Habitual patterns of emotional processing matters –Recognition of intra-individual norms is important • Emotions impede authentic decision making –Emotions impact on the coherence of preferences –Affectively first-order desires conflict with second-order desires	• Values provide a conception of the good and are essential in decision making • Minimally stable and consistent values are required for (authentic) decision making • *Pathological values* deriving from a mental disorder impede authentic decision making

Procedural account (content-neutral)	• Emotions decrease responsiveness to evidence –*Concretized emotion-belief complex* –Preoccupation with or denial of risks and benefits • Problematic emotions –Feelings of indifference concerning one’s own welfare –Pathological hopelessness • Affectively driven under- or overvaluation of risks and benefits	• Rationality of values matters • Weighing and balancing information (*reasoning*) means to assign the right value to each item of information • Values that denigrate the status of the decision maker as a person are problematic

### Domain: Emotion and Value

We have decided to consider both kinds of arguments either concerning emotion or value. It is reasonable to discuss these factors together, as they share a common feature: their evaluative function (Kluge, 2005; Charland, 2006). Both a person’s emotions and their personal values provide information about the specific valence of aspects of the world—emotions in more affective terms and values by means of more elaborated, reflective endorsement. Although emotions and values covary and overlap, it seems useful to review the literature in terms of these two categories, as in most cases either the affective dimension or the individual’s set of reflectively endorsed values is emphasized. The terms *emotion* and *value* are used here in a relatively broad sense, in line with their wide range of usage in the literature, from affective arousal to gut feelings and from more abstract values to more concrete preferences.

*Epistemic beliefs*, which also play an important role in DMC, are further to be distinguished from emotions and values. Such beliefs pertain to a person’s (mis) perception of reality or their (ir) responsiveness to evidence, as captured under the traditional *appreciation* standard. Delusion in the course of a psychotic episode is the paradigm case in which a patient’s DMC is questioned due to distorted beliefs (Grisso and Appelbaum, 1998). There is a conceptual difference between making a decision based on untrue beliefs about reality and being responsive to evidence but basing a decision on an evaluative judgment relating to those facts. Differentiating between beliefs and evaluative elements such as emotions and values is therefore important for conceptual clarity.

### Account: Procedural and Substantive

The distinction between procedural and substantive understandings of the impact of values and emotions on DMC is of fundamental importance. This relates to discussions of whether or not DMC, and more generally the concept of personal autonomy, is content-neutral (value-neutral) or content-laden (value-laden) (Freyenhagen, 2009; Owen et al., 2009).

The prevailing procedural account is characterized by its sole focus on *formal* requirements as the only reflective processes used in arriving at a decision, or on the structural character of the underlying values, preferences, and desires (Mackenzie and Stoljar, 2000). As long as these procedural demands are met, people are allowed to make decisions on whatever grounds they choose—rational or irrational. Procedural accounts are motivated by the principle of respecting pluralism in society and therefore refrain from judging the lifestyle, value system, viewpoint, or reasons underpinning a decision as more or less appropriate.

Against this, substantive accounts claim that to resist consideration of a patient’s motives is to fail to take adequate account of real conditions, as for instance in the case of irrational anxiety, so rendering DMC evaluations less meaningful (Freyenhagen and O’Shea, 2013). Here, the *content* of reasons underpinning a decision is scrutinized and judged as either problematic or unproblematic for DMC. Insofar as personal values and emotions serve as reasons, they are potentially subject to such substantive judgments, which are always value-laden in prescribing what kinds of emotions or values are acceptable. Clearly, then, substantive requirements restrict pluralism to some extent (Freyenhagen and O’Shea, 2013).

## Results

### Importance of Emotions from a Procedural Perspective

The literature deals most extensively with the impact of emotions from a procedural or content-neutral perspective. Arguments concern the necessary and appropriate extent of emotional involvement, the negative impact of emotions on cognitive faculties, the role of habitual patterns of emotional processing, and the undermining of authenticity by emotion.

#### When Patients Do Not Take Their Emotions into Account

Advocacy for the inclusion of emotions in DMC evaluations derives to a great extent from observation, and from the acknowledgment that decision making is an emotional process—that is, emotions have an essential function in decision making that adds incrementally to contributions from cognitive or analytical capacities (Loewenstein and Lerner, 2003). In support of this argument, repeated reference is made to neuroscience and to the paradigm case of patients with lesions in the ventromedial prefrontal cortex, as described at length by Antonio Damasio (Appelbaum, 1998; Charland, 1998b). Despite their fully restored intellectual capacities, these patients make disastrous decisions in complex everyday situations by virtue of their ‘hard-wired’ inability to incorporate affective cues into their decision-making process (Damasio, 1994).

The positive functions of emotions in decision making are emphasized by those who advocate stronger recognition of emotional factors in DMC evaluations. Emotions are seen to constitute a specific source of knowledge that provides us with crucial information about the overall nature of our current situation, including internal states as well as external events. They tell us about the personal value and meaning of aspects of our world and are therefore essential in generating, defining, and keeping track of our goals and preferences. Furthermore, it is argued that emotions motivate us and lend reason to our choices. In sum, they help to promote our well-being and enable us to reach value-congruent or authentic decisions (Cox White, 1994; Silverman, 1997; Charland, 1998a,b).

In light of these positive effects of emotions, it is argued that an overriding ignorance or suppression of affective cues—or a substantially impaired capacity for emotion—makes it impossible to incorporate essential information into the decision-making process. Decisions are then made without complete information, and this is considered an impediment to competent decision making (Cox White, 1994; Silverman, 1997; Charland, 1998a,b). A substantial absence of emotion is seen to constitute a loss, potentially leading to choices that are grossly detached from a person’s self and anchored solely in objective facts.

More specifically, it is argued that emotional engagement and processing is crucial to a full appreciation of one’s situation (Charland, 1998a). The argument is advanced that a merely intellectual grasp of being personally affected by a particular clinical condition and by the consequences of disease or of potential treatment options (the traditional understanding of *appreciation*) is insufficient. Alternatively, appreciation is conceived as understanding, in a more experiential sense, what the consequences would really entail—for example, “what it would be like and ‘feel’ like to be in possible future states and to undergo potential alternatives.” (Buchanan and Brock, 1989, p. 24). Indeed, it is both disturbing and dubious when a patient remains totally dispassionate and without emotion when confronted with a life-threatening disease or a serious medical decision. Mental states characterized by strong emotional avoidance, significantly impoverishing the ability to appreciate the personal relevance of information, are relevant in this regard (Rudnick, 2002). Such psychological defenses appear to play a significant role, for instance, in anosognosia and anosodiaphoria, imposing great constraints on rehabilitation (Turnbull et al., 2014). Thus, Silverman (1997, p. 171–172) argues that, for DMC, “individuals must be able to pay attention to emotions, recognize them as relevant information, remember their relationship to past and preferred states of affairs, and determine whether acting on such emotions will further their well being.” However, a caveat must be added regarding patients, who suffer from episodic memory impairment. They would be unable to think about their personal future rather than being dispassionate and without emotion. Therefore it is not the emotional factor that is lacking, but the cognitive function.

#### When Patients Are Overwhelmed by Emotions

While in the preceding argument DMC is challenged because too little attention is paid to emotional information, the reverse case is also possible and problematic. Imagine a patient who experiences intense affective arousal in the aftermath of a serious diagnosis, so that she appears overwhelmed by intense emotions, perhaps, cannot stop crying, to the extent that feelings totally overpower rational thought. Basic mental functions such as concentration, attention, or the ability to retain information are impeded to the extent that she does not meet minimal requirements for understanding and deliberation (Cox White, 1994; Banner, 2012). Such cases invite attention to emotional factors, as they impact negatively on cognitive abilities or on the traditional criteria. In general, it is worth acknowledging that cognition and emotion are strongly interwoven—in other words, that cognition inherently involves emotion (e.g., Dolan, 2002).

To conclude up to that point, DMC is concerned with the appropriate level of emotional involvement or on the inclusion of affective cues in patients’ decision-making; both a lack or an excess of emotion is problematic (Brown, 2011; Banner, 2012).

#### When Patients Process Emotions in an Atypical or Unusual Way

Imagine a patient who has been consulting a physician for years, so that her personality and preferences are well known to the physician, who is acquainted with her intuitive and emotionally driven decision-making style. On a particular occasion, however, she is unable to attend to or rely on her emotions, and behaves in a calculating and distanced way that clearly deviates from her typical conduct (Cox White, 1994). In these circumstances, concerns may arise about the patient’s DMC; the same would apply to a patient who is known to be quintessentially rational but suddenly relies only on their gut feelings. Such cases can call DMC into question because the person decides in a manner that is atypical for him or her. Abrupt changes in patients’ conduct generally warrant caution, as they indicate that something is disordered (Grisso and Appelbaum, 1998). Moreover, Cox White (1994, p. 137) argues that “Letting a patient make decisions without the evaluations he normally considers important and worthy of attention permits him to go astray from the structuring or restructuring of his life plans”; this in turn is regarded as an impediment to the patient’s well-being and autonomy of choice.

On the flip side, as long as a person complies with her typical decision-making style, it is argued that there is no reason to question her DMC even if she decides in an overly emotional or overly rational manner compared to the average. Cox White (1994), for example, holds that it is not legitimate to deem a person incompetent only because they are generally unemotional and quintessentially rational, never expressing their emotions because they do not allow themselves to experience them; to overrule a person’s typical response fails to respect his individuality. In similar vein, Mackenzie and Watts (2011a,b) discuss the implications of an emotional capacity standard for the so-called neurodiverse, such as a person diagnosed with an autism spectrum disorder, who might by such a standard be at risk of being unjustifiably deemed incompetent by virtue of their abnormal emotionality. In their view, respect for this unemotional decision-making style is warranted because it is typical for that person.

In sum, appropriate attention must be paid to person’s decision-making style. However, as the literature suggests, considerations of style and preferences might conflict in certain instances with the required minimum degree of emotional involvement as outlined above.

#### When Patients Decide Inauthentically on the Basis of Affective States

Another cluster of reasons for considering emotional factors as relevant to DMC touches on the notions of authenticity and accountability as criteria for DMC (Elliott, 1991). As Brown (2011, p. 199) observes, striking mood shifts or labile conditions seem to “undermine a person’s decision making from within as if there were no one executive decision-maker whose authority is recognized by competing mental states, as if a federation were functioning without any central government.”

When a person is affected by deep depressive feelings, for instance, their values, desires, beliefs, and dispositions are often so dramatically changed that their decisions are widely inconsistent with their authentic character in a healthy state. The patient’s choices are not truly theirs but more a result of their mental illness, and they seem not fully accountable for their decisions (Elliott, 1997). Similarly, Rudnick (2002, p. 153) argues that “pervasive emotional states or moods impact on preferences by regulating their relative weights and perhaps less commonly by generating new preferences, thus modifying the set—and hence the coherence—of preferences held by the individuals experiencing these moods.”

While regarding such incoherence as problematic for DMC, Rudnick (2002) also acknowledges the challenges posed by chronic depression or dysthymia, where changed preferences are so persistent and ingrained in personal identity that one can hardly describe them as inauthentic.

Authenticity or accountability may also be threatened in instances where, according to Frankfurt (1971), affectively driven first-order desires conflict with a person’s second-order desires. This is often observed in persons suffering from addiction and may also apply to persons with certain phobias (Frankfurt, 1971; Charland, 2002). Imagine, for example, a person who is fully aware of the benefits of treatment and is keen to have it (second-order desire) but is so terribly afraid of hypodermic needles that they take flight in panic on encountering same (first-order desire).

### Importance of Personal Values from a Procedural Perspective

In line with the prevailing procedural account, the second most elaborated cluster of arguments claims to recognize personal values in a content-neutral manner, encompassing such fundamental concerns as the indispensability of a stable set of values for decision-making in general and for DMC in particular, together with more sophisticated reasons related to authenticity.

#### When Patients Lack an Elaborated Value System

Imagine a patient suffering from advanced dementia who is increasingly losing his sense of self, including his conception of what is valuable to him and what he considers desirable to strive for. Although he can momentarily indicate his needs by showing pleasure or aversion, he is no longer able to align these responses to a represented set of personal values, making it impossible to evaluate any information about an upcoming treatment or to reflect on the factors underlying and motivating a choice. Alternatively, one may think of a 10 year-old girl who meets all the cognitive standards but is only beginning to develop a sense of her adult self and her own conception of the desirable. Again, her values may not yet be sufficiently elaborated to properly balance relevant treatment information. In both cases, DMC is challenged on the grounds that a personal value system and a related capacity to assign personal significance to one’s options are essential in decision-making. This is particularly true if competent decisions are to result in choices that are personally meaningful, based on reflections about one’s motives, and potentially beneficial for the decision-maker’s subjective well-being (Buchanan and Brock, 1989). People need a conception of the good against which they can weigh and evaluate alternative treatment options (Buchanan and Brock, 1989).

In other words, concepts of value provide reference points for reflection, explanation, and justification of one’s motivation for choosing a particular alternative. If a patient does not possess a sufficiently elaborated set of values in which their decisions are embedded, no meaningful or autonomous choices can be made, and DMC is called into question (Buchanan and Brock, 1989; Breden and Vollmann, 2004; Kluge, 2005).

#### When Patients Lack Consistent Values

The mere presence of values, however, seems insufficient. For DMC, it is additionally required that these values are minimally stable and consistent over time (Charland, 2001; Kluge, 2005; Craigie, 2013). It is argued that “sufficient value stability is needed to permit, at the very least, a decision that can be stated and adhered to over the course of its discussion, initiation and implementation” (Buchanan and Brock, 1989, p. 25)—otherwise, one can hardly develop consistent plans, and risks being caught in motivational conflicts (Kluge, 2005). Imagine a cancer patient who must decide whether he wants to continue with curative treatment that might prolong his life or to stop it and begin with palliative care. He is torn between the options and constantly changes his mind, struggling with whether to give priority to extension of life or a peaceful death and caught in a strong ambivalence that he cannot overcome by himself.

Consistency of values is closely related to the notion of authenticity, in that decisions made on the basis of enduring personal values will also appear authentic (Mackenzie and Watts, 2011b). However, it is problematic to take an enduring set of values as a criterion for either authentic or competent decision-making, given that values evolve and change over time, or may even undergo radical change in particular situations—as, for example, in drastic end-of-life circumstances (Mackenzie and Watts, 2011b; Craigie, 2013).

#### When Patients Rely on Inauthentic Values

A heated debate has arisen concerning the relation between values and authenticity in the context of mental disorders—more specifically, with reference to anorexia nervosa (Charland, 2006; Grisso and Appelbaum, 2006; Tan et al., 2006, 2009; Vollmann, 2006; Whiting, 2009). Tan et al. (2006) coined the term *pathological values* to circumscribe those values of anorexic patients that originate from their disorder rather than from the individual themselves. They argue that decisions based upon such *pathological values* challenge DMC because these values are not authentic or in line with that person’s expected values if they were not affected by a mental disorder. In the anorexic patient, for example, overvaluation of thinness may be regarded as pathological insofar as it is a causal consequence of anorexia nervosa—a mental disorder characterized by, among other things, the diagnostic criterion of intrusive fear of fatness—and so belongs to the disease rather than to the individual (Tan et al., 2006). Moreover, this overvaluation of thinness is likely to vanish when the disorder abates, and vice versa (Charland, 2006). It appears then that justifications of incompetence based on *pathological* or *inauthentic* values are closely bound to or apply only in the context of a diagnosed mental disorder (Tan et al., 2009).

As previously discussed in the context of affective disorders and authenticity, challenges regarding this argument arise when anorexic patients experience an enduring change of personal identity, where the disorder becomes so constitutive of self that patients can no longer imagine being free of it (Tan et al., 2006; Hope et al., 2013). Intriguingly, Hope et al. (2011) have shown that many patients suffering from anorexia nervosa experience the disorder as separate from the real self, distinguishing between an *authentic real* self and an *inauthentic anorexic* self. However, others experience their anorexia “not as a separate self but as integral to a single self.” (Hope et al., 2011, p. 24), calling into question whether we can still speak of inauthentic values in these cases.

### Importance of Emotions from a Substantive Perspective

A third cluster of arguments concerns the substantive or content-laden influence of emotions on DMC. Interestingly, most of these contributions do not pertain directly to the content of emotions but argue for their indirect impact on substantive elements by way of epistemic beliefs. Insofar as such beliefs rely upon epistemic norms that are content-laden, the manner of their constraint by emotion is best discussed from a substantive perspective. By contrast, there are no explicit and profound analyses or justifications of the appropriateness of specific emotive reasons in the context of DMC. There are only some theoretical reflections, especially in relation to depression, that more or less implicitly allude to the relevance of emotions from a substantive point of view.

#### When Patients Misperceive Reality Due to Affective States

Emotions are seen to impact negatively on DMC by affecting the patient’s responsiveness to evidence, their perception of reality, or their epistemic beliefs, respectively. In terms of the traditional approach, one might say that affective factors impact negatively on the *appreciation* criterion.

An elaborated account on the impact of emotion on epistemic beliefs is presented by Halpern (2011, 2012), who labels the DMC-impoverishing interplay she describes as a *concretized emotion-belief complex*. At the core of her argument lies the observation that there are patients who are caught in an affective state—often in the aftermath of a traumatizing or shocking event—that is frequently accompanied by catastrophic thinking, rendering them unable to feel differently in the present or to imagine feeling differently in the future. In addition, they are unable to grasp that they are subject to this emotional point of view and that their current affective state directly impacts on their epistemic beliefs about the future. As a consequence, their cognitive responsiveness to evidence is undermined, as is their ability to deliberate meaningfully on the future (Halpern, 2012).

Similarly, Meynen (2011) argues that in depression a person’s perception of the world and of their possibilities certainly changes, even to the point of substantial bias that would be considered problematic for competent decision-making. As Bursztajn et al. (1991, p. 386) note, affective disorders are often characterized by “an emotionally involving, self-convincing preoccupation with the risks of treatment coupled with denial of the benefits.” It is the profound feeling of hopelessness that can render the depressed patient incapable of envisioning any possibility of recovery or even of relief from suffering (Leeman, 1999).

Comparable effects have been observed and discussed in the context of anorexia nervosa, whose characteristic signs include strong anxiety associated with eating to put on weight and related negative feelings such as self-disgust on seeing one’s body. These feelings lead anorexic patients to a judgment that they are too fat, which is counterevident from an objective point of view. Although often able to see and intellectually grasp the objective evidence, their affective responses prompt these patients to believe differently and to assess their weight against a purely subjective standard (Hope et al., 2013). In short, they lack affective responsiveness to objective norms and evidence.

#### When Patients Rely on Problematic Emotions

Proponents of a substantive account of DMC place particular emphasis on the reasons underlying a patient’s choice, and on the judgment of these reasons as more or less appropriate or supposedly *recognizable* (Freedman, 1981; Charland, 1998b; Banner, 2012, 2013). It is further acknowledged that emotions constitute a particular class of reasons and are therefore subject to such evaluation.

Depression is an affective disorder characterized by hopelessness, helplessness, deeply entrenched feelings of guilt and worthlessness, anhedonia, and other symptoms. These feelings are likely to affect the value assigned by patients to themselves, to their lives, and more specifically to the treatment options and outcomes they face. One likely consequence of such feelings is that a patient will become largely insensitive to their own welfare, perhaps to the point of not caring about risk, or even positively valuing negative consequences such as death (Elliott, 1997; Freyenhagen and O’Shea, 2013). That being so, should we ever consider someone’s deep feelings of worthlessness as a legitimate or *recognizable* reason to refuse life-saving treatment? Bursztajn et al. (1991, p. 384) argue that “affective disorders may impair DMC in a detectable and identifiable way, primarily influencing the meaning and weight given to treatment risks and benefits.” And Rudnick (2002) points to felt indifference and the problem of undervaluing positive outcomes in depression—both of which, he says, undermine DMC. Other authors only touch on these substantive features; Sullivan and Youngner (1994), for example, point to the difficulty of distinguishing between realistic and pathological hopelessness among terminally ill patients.

By the same token, most of these authors are also aware of the challenge inherent in substantive approaches in terms of the value judgment they entail: “Assessing a patient as emotionally incompetent may bring us once again into the difficult realm of distinguishing the competency assessor’s values or preferences for treatment from the patient‘s emotional state.” (Glass, 1997, p. 22). Halpern (2011, 2012) takes an explicit position that declines to deem a depressive patient incompetent if they undervalue life. According to her, as long as the depressed person retains their cognitive abilities and is responsive to evidence, his or her values and choices should be respected.

### Importance of Personal Values from a Substantive Perspective

In the realm of values, analyses of their relation to epistemic beliefs (as in the context of emotions) do not exist, or values and beliefs are wrongfully equated (e.g., Grisso and Appelbaum, 2006). Moreover, the predominant argument in the literature concerns why the content of values matters, but in-depth analyses of specific types of values remain rare.

#### When Patients Rely on Problematic Values

From a substantive perspective, personal values are considered relevant to DMC in that they provide reasons on which to base decisions. As such, they are subject to normative judgments regarding their appropriateness. Charland (2001, p. 139), for example, states that “simply having values is not enough. A certain kind of rationality is also required.” He stresses that normative considerations associated with value can never be eliminated from the evaluation of DMC. Imagine, for instance, a man suffering from a disease requiring hospitalization and treatment for at least 2 weeks. He refuses treatment, explaining that he does not want to stay away from home for so long, leaving his precious plants behind. He accepts the potential health impairments as long as he can stay with his many plants—his dearest companions. In such a case, discussion may proceed from whether or not his reason to forego treatment is sufficiently *recognizable* or rather indicative of incompetence.

Other authors are more specific, arguing that judging a person’s capacity to use, weigh, and balance information—termed *reasoning* in the traditional approach, where it is regarded as a purely procedural standard—necessarily involves a normative judgment (Banner, 2012, 2013; Holroyd, 2012). In saying that a person is properly weighing and balancing information, we refer not only to the logical consistency of their argumentation but also to whether the person assigns to each item of information its due significance for the decision-making process, or weighs the information appropriately. Accordingly, Holroyd (2012, p. 157) provocatively asks whether “weighing information requires that certain specific commitments and rankings are held?” In the context of anorexia nervosa, one could then say, for instance, that the patient is “improperly valuing nourishment and giving too much weight to food avoidance and maintaining a low weight.” (Holroyd, 2012, p. 157) From this perspective, we would then speak of pathological values because these are inherently problematic rather than merely impeding authentic decisions (Vollmann, 2006).

A more concrete stance on why particular values are problematic is taken by Kluge (2005, p. 299). He suggests that values that “denigrate the status of the decision-maker or others *as person*”—and therefore inherently conflict with principle-based values that acknowledge the equal and intrinsic moral worth and dignity of human beings, as proclaimed in the Universal Declaration of Human Rights—challenge competent decision making. As an example, he cites the octogenarian who foregoes life-saving treatment because she perceives herself to cause a burden as an unproductive member of society and of her family.

Proponents of a substantive understanding of DMC are entirely aware of the well-known risks of arbitrariness and undue paternalism in making value judgments (Breden and Vollmann, 2004; Tan et al., 2006; Whiting, 2009; Radoilska, 2012) and try to find ways of dealing with the implied challenges (Freedman, 1981; Banner, 2013; Banner and Szmukler, 2013; Freyenhagen and O’Shea, 2013). It goes beyond the scope of this paper to provide a full review of existing proposals for dealing with these problems or identifying a solution. However, in line with Freyenhagen and O’Shea (2013), we believe that increased transparency or explicit discussion of the values and beliefs guiding DMC evaluations, as well as democratic contestability of conditions for DMC and inter-subjective validation of judgment, can help to reduce the risk of arbitrariness.

## Discussion And Conclusion

In this paper, the literature on the role of emotions and personal values in medical DMC has been reviewed. Without offering a detailed normative justification for the inclusion of emotions or values in DMC evaluations, the overview provides a summary of arguments and justifications advanced elsewhere. To that end, these arguments are clustered in four categories, from most to least elaborated, bringing conceptual clarity and identifying potential research gaps. In this way, it contributes to the ongoing debate by suggesting starting points for future research to delve more systematically into specific concerns, placing a stronger emphasis also on clinical correlates. The following discussion shall present some broader reflections on the specific challenges concerning the role of emotions and values in DMC evaluations.

### Overlapping Themes

The literature on the subject proves to be quite extensive, and provides a variety of reasons for including emotion or value in DMC evaluations. Furthermore, a closer look at these arguments reveals the complex nature of DMC. Although we have tried to sort the literature within a structuring framework in the interests of more conceptual clarity, things might not be as clear-cut in reality, given the overlap between emotion and value and the interplay between cognition and emotion, and where a distinction between procedural and substantive factors is not always easily established.

This overview shows that similar themes emerge in the realms of emotion and value. Both are relevant as indispensable elements in decision making—or, indeed, as a potential threat to the authenticity or accountability of a decision. Furthermore, consistency over time plays a role, in terms of a habitual pattern or style of emotional engagement on the one side and a minimally stable set of values on the other side. Moreover, both emotion and value figure as reasons underpinning a decision that can be more or less appropriate in certain circumstances. As already mentioned, there is also a conceptual overlap; emotions seem to include a valuational component, and rationally endorsed values are likely to be accompanied by some emotional involvement. As a consequence, we may attribute DMC undermining aspects to either domain, of emotion or value. In the context of anorexia nervosa, for example, the prevailing discourse speaks of *pathological values* (Tan et al., 2006), but a few prefer to talk about *pathogenic affective states* (Charland, 2013). In respect of other disorders, such as depression, the opposite seems to hold, as a majority argues from the point of view of emotions.

### Different Possibilities for Interpretation

In general, it seems that same phenomena can be interpreted from different angles. To illustrate this, let us revisit the patient who is afraid of hypodermic needles to such an extent that he refuses treatment against his better knowledge and intent. This case was discussed in relation to Frankfurtian second- and first-order desires, and so was conceived an issue of authenticity or accountability (Frankfurt, 1971). However, one could equally argue otherwise, by demonstrating that this anxiety is so strong that it totally overwhelms rational thought, or cognitive abilities—or otherwise again, by judging it from a substantive perspective and arguing that the anxiety is so irrational that we cannot accept it as an appropriate reason to forgo treatment.

All of these arguments appear somehow plausible, so that questions arise, first, about which of these arguments provides the right justification for an incompetence judgment, and second, which of them reflects our actual reason for doubting the patient’s DMC. It seems unlikely that univocal answers to these questions can be obtained but rather that all of these aspects together, reflecting the specificity and complexity of the case, cause us to question DMC and help us to justify an incompetence judgment.

### Case-Specificity

The specificity and complexity of cases therefore seem important, and this is supported by other observations from the literature. Issues of value stability, emotional involvement and authenticity-impoverishing impacts of emotions and values all require more or less subtle differentiation to be indicative for incompetence: A certain degree of value stability is necessary, but people should also be allowed to change values radically; emotional involvement that is too weak or too strong appears indicative of incompetence, but this depends on the extent to which cognitive abilities are affected, or on the person’s typical decision-making—even, perhaps, on the causes for inappropriate emotional engagement. An inability to incorporate affective cues may be differently interpreted in the context of precisely discernible brain damage than in the context of a neurodiverse condition such as autism. Furthermore, the authenticity or accountability argument seems to work particularly well in the context of a diagnosed mental disorder that implies a distinction between a diseased inauthentic and a healthy authentic self. There again, however, no universal claims apply, as it seems necessary to differentiate between the acutely and chronically diseased. Finally, the substantive account of emotions and values appears particularly case-sensitive, even to the point of being accused of total arbitrariness.

The absence of precise criteria concerning emotion and value in DMC evaluations is unsurprising, as such criteria could be formulated only conditionally and with an undefined number of *if* provisions. It appears, then, that emotions and values cannot be judged in isolation but only in light of their interaction with each other and with other factors. In place of predefined, rigid criteria, a more flexible, context-specific approach is clearly required.

### Reconciliation Efforts

The difficulty to formulate criteria in terms of emotions or values seems to contrast with the current traditional approach, which purports to give well-defined, universally applicable, clearly operationalized and objectively verifiable criteria. In this light, efforts to take into account more ambiguous and complex soft factors such as emotion and value appear to represent a great threat for proponents of the traditional approach, who usually defend their view and argue against emotional or valuational criteria by presenting various arguments.

In the first place, they may refer to the authority of the U.S. law from which the traditional criteria are derived, arguing that modifications of the legal doctrine require rigorous proof of the need for other criteria, as well as further consideration of the costs involved in changing the legal practice (Appelbaum, 1998; Grisso and Appelbaum, 2006). On the other hand, they may argue that the traditional criteria in fact capture those patients who are presumably lacking emotional or valuational capacity (Grisso and Appelbaum, 2006), and that some non-cognitive factors are already implicit in the traditional account (Kim, 2010). For instance, in the debate on *pathological values* in anorexia nervosa, Grisso and Appelbaum (2006) argue that the traditional *appreciation* criterion already includes some consideration of values. To establish this link, they equate values with epistemic beliefs: “In fact, the analysis of most cases of lack of appreciation find values, applied in a manner that involves a distortion of reality, at the heart of the matter” (Grisso and Appelbaum, 2006, p. 295). They then illustrate the range of distorted beliefs underpinning the decisions of anorexic patients—for example, their belief that they are fat, although in reality they are just skin and bones. Certainly, these beliefs challenge DMC, but they provide a justification that differs from judging the value of thinness as problematic for DMC because it can hardly reflect an authentic value in the context of anorexia nervosa. It seems, then, that Grisso and Appelbaum (1998) merged the notions of value and belief to bring discussion of *pathological values* back within the confines of the traditional approach.

A similar mechanism can be observed with regard to the notion of *appreciation*, one of the four traditional standards clearly defined and operationalized by Grisso and Appelbaum but also occurring in ordinary language, with quite ambiguous and versatile meanings. Authors may speak of an appreciation of consequences and mean quite different things; for some, it means being responsive to evidence, while for others it refers to the ability to emotionally grasp the personal significance of the consequences, and for still others it implies an allocation of proper weights to each consequence. Thus, ostensibly operating within the confines of the traditional approach, it is actually about value and emotion. Therefore, we advocate for more explicit mention and discussion of these non-cognitive factors to prevent potential misunderstandings and conceptual confusions, and to enable more targeted interventions in support of patient’s decision making.

### Operationalizing and Measuring Emotions

Opponents of an emotion-inclusive approach to DMC further argue that emotions need to be clearly operationalized and measurable in a reliable manner before being included in DMC evaluations (Appelbaum, 1998). This is indeed a challenging or even conclusive argument, as it seems more difficult as well as inappropriate to provide technical operationalizations and measurements for emotions comparable to those used in the assessment of cognitive faculties (Kontos et al., 2015). Emotional processing seems not only more subjective but also to require more contextually embedded evaluation that is sensitive to the person in their entirety. Moreover, relational aspects such as empathy seem to play a crucial role in the perception and evaluation of emotional components—it is rather about understanding patients than measuring them. In this light, a rethinking of assessment procedures to take proper account of emotional factors seems worthwhile.

### Dealing with Different Requirements

To conclude, there seems to be a tension between the desire to retain some legally binding criteria—universally applicable, value-neutral, concise, and slight as possible—that can serve as points of reference, especially in the case of legal proceedings, and the requirement to take proper account of the complexity and specificity of single cases—the nuanced occurrences, interplays, and differentiations of mental processes, and their embedding in normative frames which together touch on our intuitions to challenge a person’s DMC.

It is open to discussion how to reconcile these two requirements and to balance the advantages and disadvantages of a minimalistic and a more comprehensive and case-specific approach. Nevertheless, it appears that we currently tend to interpret the existing standards too narrowly, even try to adhere desperately to them (as the above-mentioned reconciliation efforts show), and sometimes forget that there is actually a scope of discretion giving latitude to the peculiarities of individual cases. After all, the traditional criteria were inferred from case law, perhaps too directly translated into standardized measures, so that in the course of this translation the case-based approach was inappropriately replaced by a standardized frame into which each case subsequently had to fit.

### Directions for Future Research

We understand the literature on emotion and value to be an attempt to explore the scope of discretion and peculiarities applying to single or groups of cases, and to provide a nuanced and case-sensitive understanding of the factors that cause us to question a person’s DMC. The formulation of additional operationalized criteria seems not to be—and, in light of the aforementioned difficulties, cannot be—the primary purpose of these contributions. Nonetheless, these analyses are crucial, as they help to sensitize those who must evaluate and justify incompetence to the multifold and case-related interplays between contextual factors, various mental faculties, and their normative underpinnings.

In general, a greater emphasis on case analysis seems promising for a still more nuanced understanding of DMC (Owen et al., 2009). This would include a stronger focus on patient narratives, allowing relevant observations and statements to be contextualized in order to fully understand and properly interpret and appraise them. In particular, conclusions about the authenticity of a decision or the *recognizability* of underlying reasons require empathic immersion and analysis of patients’ stories and experiences. Continuative qualitative analyses of the nature and experience of anorexia nervosa are exemplary in this regard (Charland et al., 2013).

According to the proposed framework, substantive accounts of both emotions and values will need to be investigated, as these are relatively underrepresented in current analyses (see also **Table 1**). Of particular interest is the deeper analysis of fine differences between merely eccentric values, emotions, or reasons in general and those that are problematic for DMC, and the moderating conditions that determine the transition. This would include further consideration of how to conceive of and deal with the related problems of unjustified paternalism and arbitrariness, as well as further reflection on framing effects arising from diagnosis of a mental disorder. While the healthy eccentric is explicitly granted the right to self-determination, the eccentric with a psychopathology will probably be required to present as more “normal” than those without a psychiatric diagnosis if they are to be deemed competent.

## Author Contribution

The paper has arisen from a broader research project that investigates DMC from an empirical and conceptual perspective. All authors contributed significantly to the design of this project, participated in questionnaire design (for the empirical part), data interpretation, and discussion of the overall results and of the findings related to emotions. HH has conducted the literature search and extraction of arguments, and has written up the paper. All authors have critically revised several consecutive versions of the draft manuscript and approved the final version.

## Conflict of Interest Statement

The authors declare that the research was conducted in the absence of any commercial or financial relationships that could be construed as a potential conflict of interest.
